# Differential Urinary Microbiota Composition Between Women With and Without Recurrent Urinary Tract Infection

**DOI:** 10.3389/fmicb.2022.888681

**Published:** 2022-05-26

**Authors:** Lei Huang, Xiangyan Li, Bo Zheng, Pengtao Li, Dali Wei, Chenwei Huang, Liying Sun, Haixia Li

**Affiliations:** ^1^Department of Clinical Laboratory, Peking University First Hospital, Beijing, China; ^2^Department of Anti-infection, Institute of Clinical Pharmacology, Peking University First Hospital, Beijing, China; ^3^Beijing Yitong Qijun Technology Co., Ltd., Beijing, China

**Keywords:** recurrent urinary tract infection (RUTI), urinary microbiota, 16S rRNA, next generation sequencing (NGS), standard urine culture

## Abstract

**Background:**

Recurrent urinary tract infection (RUTI) is common and burdensome in women. Due to the low concentration or slow-growing of uropathogens in RUTI, standard urine cultures (SUCs) are often negative. Next-generation sequencing (NGS) of bacterial 16S rRNA gene is more sensitive and could be used to reveal the differential microbiota between patients with RUTI and asymptomatic controls.

**Methods:**

Women (aged ≥ 18 years) with clinically diagnosed RUTI with negative SUC and age-matched women asymptomatic controls with normal urinalysis were enrolled. Their midstream voided urine specimens were collected and processed for NGS (Illumina MiSeq) targeting the bacterial 16S rRNA gene V3-V4 region. The dataset was clustered into operational taxonomic units (OTUs) using QIIME. Taxonomic analysis, alpha diversity, beta diversity, multivariate statistical analysis, and linear discriminant analysis effect size (LEfSe) for differential analysis were performed and compared between patients with RUTI and asymptomatic controls.

**Results:**

A total of 90 patients with RUTI and 62 asymptomatic controls were enrolled in this study. Among them, 74.4% (67/90) and 71.0% (44/62) were successfully amplified and sequenced their bacterial 16S rRNA gene. In the alpha diversity analysis, the chao1 index and observed species index were significantly lower in the RUTI group than in the control group (*P* = 0.015 and 0.028, respectively). In the beta diversity analysis, there was a significant difference between the 2 groups [Analysis of similarities (ANOSIM), *R* = 0.209, *P* = 0.001]. The relative abundance of 36 bacterial taxa was significantly higher, and another 24 kinds of bacteria were significantly lower in the RUTI group compared with the control group [LEfSe analysis, *P* < 0.05, linear discriminative analysis (LDA) score > 3], suggesting that *Ralstonia*, *Prevotella*, *Dialister*, and *Corynebacterium* may play an important role in RUTI.

**Conclusion:**

The urinary microbiota of women with clinically diagnosed RUTI were significantly different from age-matched asymptomatic controls.

## Introduction

Urinary tract infection (UTI) is one of the most common infections worldwide, especially in women. This is thought to be due to women’s physiological structure and hormonal status. UTIs have high incidence rates and cause an economic burden (Flores-Mireles et al., 2015; Klein and Hultgren, 2020). According to a study from the United States, over 60% of women experienced at least one UTI during their lifetime, and 20–30% of them experienced recurrent UTI (RUTI) within the next 6 months (Foxman et al., 2000). RUTI is defined as the occurrence of ≥ 2 symptomatic episodes of UTI within 6 months or ≥ 3 episodes within 12 months (Kranz et al., 2018). RUTI significantly reduces the quality of life and leads to more medical consultations per year. It is highly prevalent, costly, and burdensome among women of all ages and races (Wagenlehner et al., 2018).

Microbial confirmation by urine culture is important in establishing the diagnosis of RUTI, and further antibiotic susceptibility testing provides tailoring of therapy (Anger et al., 2019). Patients with RUTI are frequently exposed to previous antibiotic treatment. Their bacterial concentration may be low in urine due to the antibiotic treatment. The bacteria of low concentration or slow-growing uropathogens may be missed by standard urine culture (SUC). The negative urine culture results have posed a challenge for antimicrobial therapy.

Bacterial 16S rRNA amplicon sequenced by the high-throughput next-generation sequencing (NGS) technology demonstrates that urine is not sterile, and contains diverse microbiota in both men and women (Nelson et al., 2010; Dong et al., 2011; Fouts et al., 2012; Wolfe et al., 2012). NGS is more sensitive than SUC. McDonald et al. (2017) showed that 100% of symptomatic patients with UTI had NGS positive test results for urinary bacteria, compared with a positive rate of 30% in SUC. Burnett et al. (2021) found that expanded quantitative urine culture (EQUC) could detect more urinary microbes than SUC, and their urobiome compositions were associated with distinct clinical profiles in patients with RUTI. Hochstedler et al. (2022) showed that EQUC detected more uropathogens than SUC from catheterized and voided urine in patients with RUTI. Urinary microbiome detected by NGS in health and disease has been characterized in recent years (Lewis et al., 2013; Pearce et al., 2014; Pohl et al., 2020). The studies on urinary microbiota of women patients with RUTI by NGS are still limited. However, it is a rapid growing field. A consensus to advance urobiome research was formed recently (Brubaker et al., 2021).

Peking University First Hospital is the 1,800-bed tertiary general hospital of Peking University, Beijing, China. Both nephrology and urology departments are the top 1 key discipline in China. Patients with RUTI with negative SUC were usually found in outpatient (Monsen and Ryden, 2017). We have assessed a small group of 10 patients with RUTI with a positive SUC. All of them were a pure culture of single uropathogen ≥ 10^4^ CFU/ml, which was a common circumstance in positive urine culture. The results are in good agreement between urine culture and NGS. Therefore, physicians can treat these patients based on urine culture and antibiotic susceptibility results. NGS does not show an advantage in these patients considering its high cost and complex procedure. The real clinical challenge is the patients with RUTI with negative urine culture, which could show the advantage of NGS. Thus, in this study, we only compared the urinary microbiota between patients with RUTI with negative SUC and age-matched asymptomatic controls.

## Materials and Methods

### Subjects and Study Design

Women outpatients (aged ≥ 18 years) with a clinical diagnosis of RUTI from January 2021 to May 2021 were included with the following criteria: (1) clinically diagnosed RUTI, which was ≥ 2 symptomatic episodes of UTI within 6 months or ≥ 3 episodes within 12 months; (2) around 72 h before enrollment, there were ≥ 2 signs or symptoms, namely, fever (≥ 37.3°C), dysuria, frequent urination, urgent urination, painful urination, suprapubic pain with tenderness, backache, tenderness of costal angel, or percussion pain of renal area; and (3) urinalysis of white blood cell > 5/high power field (HPF). The exclusion criteria were as follows: (1) patients with antibiotic treatment in the previous 48 h and their symptoms relieved; (2) patients with kidney transplantation; (3) patients with urinary intubation; and (4) patients during pregnancy or lactation periods.

Age-matched women asymptomatic control subjects were enrolled during the same period. These were: (1) women (aged ≥ 18 years) without any signs or symptoms of UTI in the past 1 year, (2) with normal urinalysis results, (3) no prior antibiotic usage within 1 month, (4) no urology or kidney abnormality or history of urological surgery, (5) no pregnancy, and (6) no sexually transmitted infections. They were randomly selected during health examination in our hospital. Their informed consents were signed. The required information was obtained by laboratory reports and telephone follow-up.

This study was approved by the ethics committee of Peking University First Hospital (approval number: 2021-191), and informed consent was obtained from each participant.

### Specimen Collection

Approximately 30–50 ml midstream voided urine samples of both patients with RUTI and asymptomatic controls were collected by following standard “clean catch” protocol with the guidance of a physician to avoid contamination to the great extent (China Health Professional Standards, 2016). The procedure requires that the vulva must be cleaned and separated when collecting urine. The urine specimens were sent to Clinical Microbiology Laboratory within 2 h using sample transfer box with an ice bag to keep the temperature of 0–4°C. SUC was performed by following the Standard Operation Protocol (SOP) from the Health Professional Standard of China (China Health Professional Standards, 2016). The remaining specimens were stored at –70°C freezer as soon as possible to avoid RNA degradation. All the stored urine specimens were sent to Allwegene Company (Beijing, China) for NGS in one batch to avoid batch-to-batch variation.

### DNA Isolation and 16S rRNA Amplicon Sequencing

Of note, 30 ml of clean catch mid-stream urine was centrifuged at 10,000 *g* for 30 min. Pellets were used for DNA extraction, following the protocol of DNeasy Blood and Tissue Kit (Qiagen, Hilden, German) in the biological safety cabinet to avoid contamination. The concentration and quality of extracted DNA were checked at A260/A280 on a NanoDrop (Thermo Fisher Scientific, Waltham, United States). According to the concentration, DNA was diluted to 1 ng/μl using sterile water.

Bacterial 16S rRNA gene of distinct V3-V4 hypervariable region was amplified by PCR with specific primers 338F (5′-ACTCCTACGGGAGGCAGCAG-3′) and 806R (5′-GGACTACHVGGGTWTCTAAT-3′). The barcode sequences were added to the 5′ end of the forward and reverse primers. The barcodes and primers were provided by Allwegene Company (Beijing, China). All PCR reactions were carried out with Phusion High-Fidelity PCR Master Mix (New England Biolabs, Ipswich, United States). PCR used 50 μl of reaction volume, containing 2.5 μl forward primer (10 μM), 2.5 μl reverse primer (10 μM), 25 μl 2 × Phusion Master Mix, 2 μl template DNA, and 18 μl nuclease-free water. Thermocycling conditions were as follows: 98°C for 30 s, followed by 30 cycles of 98°C for 10 s, 55°C for 30 s, and 72°C for 15 s with a final extension of 72°C for 10 min. The PCR products were purified with Qiagen Gel Extraction Kit (Qiagen, Hilden, Germany). The experiments were performed according to the manufacturer’s instructions with no modification. Both positive and negative controls were included during DNA isolation and 16S rRNA gene sequencing.

Deep sequencing was performed on the MiSeq platform (Illumina, United States) at Allwegene Company (Beijing, China). The pooling and quantification of library were performed. The sequencing protocol was paired-end with the targeting length of 450–500 bp. Image analysis, base calling, and error estimation were performed using the Illumina analysis pipeline (version 2.6).

### Bioinformatic Analysis

Raw data were screened and removed from consideration if they were shorter than 230 bp, had a low quality score (≤20), contained ambiguous bases, or did not exactly match primer sequences and barcode tags. Qualified reads were separated using sample-specific barcode sequences and trimmed with Illumina analysis pipeline (version 2.6). The barcode and primer were removed from raw data and spliced into raw tags.

For quality control, the raw reads were first processed using Trimmomatic (version 0.36) and Pear (version 0.9.6). Reads containing “N” or with quality less than Q20 were filtered. Then, the clean reads were merged into tags by Flash (version 1.20) and Pear (version 0.9.6), with a minimum overlap of 10 bp and a maximum false match rate of 0.1. “unoise3” from the usearch10 software was also used to de-noise the data. The known chimeras were removed by searching the SILVA gold database, and the *de novo* chimeras were removed employing uchime3.

The sequences were clustered into operational taxonomic units (OTUs) using the USEARCH (version 10) software with the sequence similarity ≥ 97%. Rarefaction curves were generated and microbial diversities were estimated. The Ribosomal Database Project (RDP) Classifier tool (version 2.2) was used to classify all sequences into different taxonomic groups. The SILVA (version 128) database was used to assign taxonomy.

The alpha diversity indexes (e.g., chao1, Shannon, observed species, and PD whole tree) were calculated using QIIME version 1.8.0. To examine the similarity between different samples, clustering analysis and principal component analysis (PCA) were used based on the OTU information from each sample using the R software (version 4.1.2). The beta diversity between each group was compared using the relative abundance of the species in each sample, rather than the OTU table. In this step, the subsampled data were uniform. The evolutionary distances between microbial communities from each sample were calculated using the Bray–Curtis algorithms and represented as an Unweighted Pair Group Method with Arithmetic Mean (UPGMA) clustering tree describing the dissimilarity (1-similarity) between multiple samples (Jiang et al., 2013). A Newick-formatted tree file was generated through this analysis. To compare the membership and structure of communities in different samples, heat maps were generated with the top 20 most abundant OTUs using mothur (version 1.44.2).

Taxonomic analysis, alpha diversity, beta diversity [partial least squares discrimination analysis (PLS-DA)], multivariate statistical analysis, and linear discriminant analysis effect size (LEfSe) (version 1.1.01) (Segata et al., 2011) for differential analysis were performed and compared between RUTI and asymptomatic controls.

### Statistical Analysis

The continuous variables were presented as “mean ± standard deviation” when they were normal distribution. If they were not a normal distribution, they were presented as “median and quartiles.” The categorical variables were also expressed as absolute numbers. The statistical comparison between RUTI and control was evaluated using Student’s *t*-test for continuous variables (e.g., age) with normal distribution and Pearson’s chi-square test for categorical variables using the SPSS software (version 22.0). The statistical significance was defined as *P* < 0.05 with two sides.

## Results

The demographic characteristics of patients with RUTI and age-matched asymptomatic controls are summarized in Table 1. No significant differences were observed in age, NGS-positive (with successful sequencing result) rate, diabetes, and menopause status between the 2 groups (*P* > 0.05). The 16S rRNA gene sequencing detected urinary bacteria in 74.4% (67/90) of patients with RUTI and in 71.0% (44/62) asymptomatic controls. A total of 67 patients with RUTI and 44 asymptomatic controls with successful 16S rRNA gene sequencing results were processed for microbiota analysis.

**TABLE 1:** The demographic characteristics of included patients with RUTI and asymptomatic controls.

	RUTI (*N* = 90)	Asymptomatic controls (*N* = 62)	*P*-value
Age (years)	49.5 ± 15.2	46.0 ± 11.7	0.134
NGS-positive	74.4% (67/90)	71.0% (44/62)	0.635
Diabetes	10.0% (9/90)	4.8% (2/62)	0.113
Menopause status	57.8% (52/90)	46.8% (29/62)	0.181
Urology or kidney abnormality	8.9% (8/90)	0.0% (0/62)	N/A*
Prior antibiotic usage within 1 months	27.8% (25/90)	0.0% (0/62)	N/A
History of urological surgery	21.1% (19/90)	0.0% (0/62)	N/A
**Urinalysis abnormality**			
Urine WBC abnormality	37.8% (34/90)	0.0% (0/62)	N/A
Urine RBC	43.3% (39/90)	0.0% (0/62)	N/A
Urine bacteria	24.4% (22/90)	0.0% (0/62)	N/A
Urine nitrite	0.0% (0/90)	0.0% (0/62)	N/A

WBC, white blood cell; RBC, red blood cell; NGS, next-generation sequencing. *N/A: Characteristics unavailable for healthy controls.

**FIGURE 1:**
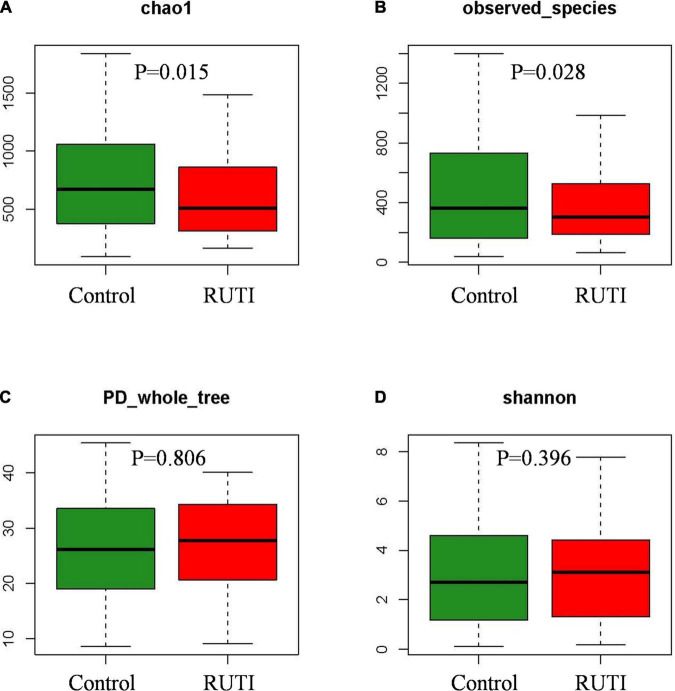
Alpha diversity indices Chao1 **(A)** and observed species **(B)** significantly decreased in the RUTI group compared with the control group. PD whole tree **(C)** and Shannon **(D)** showed no statistical differences (Kruskal–Wallis test, *P* < 0.05).

The high-quality clean tags were clustered into a total of 4,390 OTUs. Alpha diversity analysis is shown in Figure 1. Chao1 index and observed species indexes were significantly different between the 2 groups (*P* < 0.05). The two indexes reflect the richness of species, without considering the relative abundance of each species. Thus, they are easier to show the existence of low abundant species. Shannon and PD whole tree indexes reflect the diversity of species, which are affected by the species’ evenness and richness.

**FIGURE 2:**
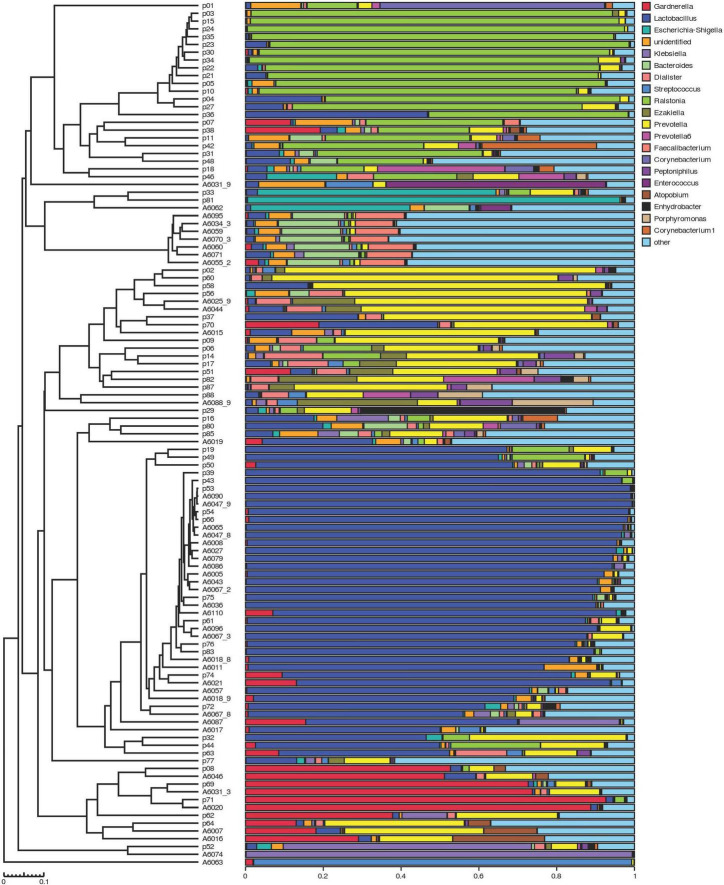
The clustering histogram of individual sample at the genus level. Hierarchical clustering analysis between samples is shown on the left. Histogram of individual sample’s community structure is shown on the right. X-axis was the relative abundance of bacteria, and Y-axis was the sample name (p stood for patients with RUTI, A stood for control).

**FIGURE 3:**
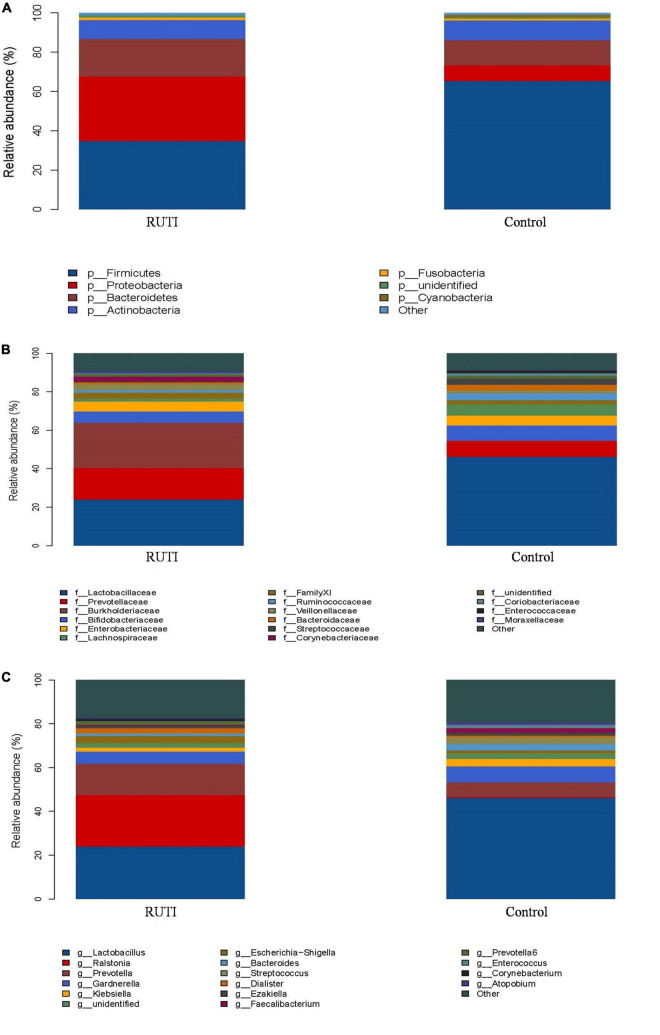
Relative abundance bar plot of phylum **(A)**, family **(B)**, and genera **(C)** in the RUTI and control groups.

The clustering histogram of an individual sample at the genus level is shown in Figure 2, which showed the communities in each sample. The relative abundance between RUTI group and control group at phylum, family, and genus levels is illustrated in Figures 3A–C. The top 3 dominant phyla were as follows: *Firmicutes* (relative abundance: 34.8% in RUTI vs. 65.4% in control), *Proteobacteria* (32.8% in RUTI vs. 8.02% in control), and *Bacteroidetes* (19.1% in RUTI vs. 12.8% in control). The top 3 dominant genera were as follows: *Lactobacillus* (24.0% in RUTI vs. 46.3% in control), *Ralstonia* (23.5% in RUTI vs. 0.242% in control), and *Prevotella* (14.4% in RUTI vs. 6.82% in control).

**FIGURE 4:**
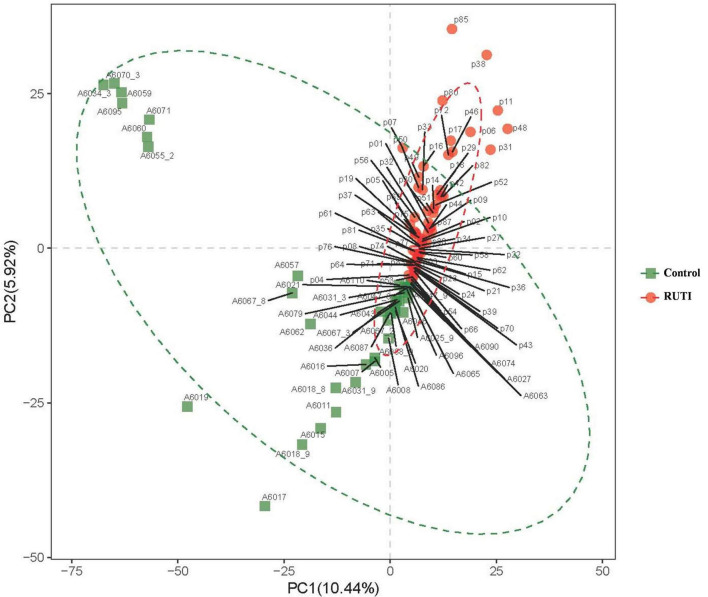
Beta-diversity indices revealed by Partial Least Squares Discrimination Analysis (PLS-DA), ANOSIM, *R* = 0.209, *P* = 0.001. PC1 and PC2 in the X/Y-axes represented the two principal dimensions that can reflect the largest variance.

In the beta diversity analysis, inter-group difference evaluated by PLS-DA is shown in Figure 4. There was a significant divergence between RUTI and control groups (ANOSIM, *R* = 0.209, *P* = 0.001), indicating the difference of inter-group was larger than intra-group.

**FIGURE 5:**
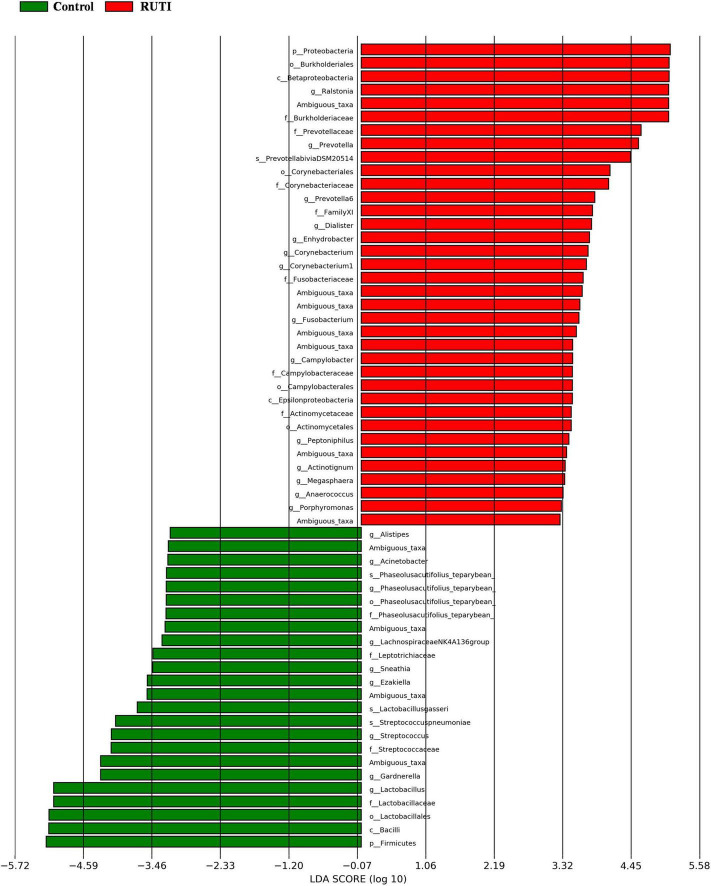
Differentially abundant bacteria between RUTI and controls, revealed by linear discriminant analysis effect size (LEfSe) analysis, with the bacteria of *P* < 0.05 and LDA score > 3.

**FIGURE 6:**
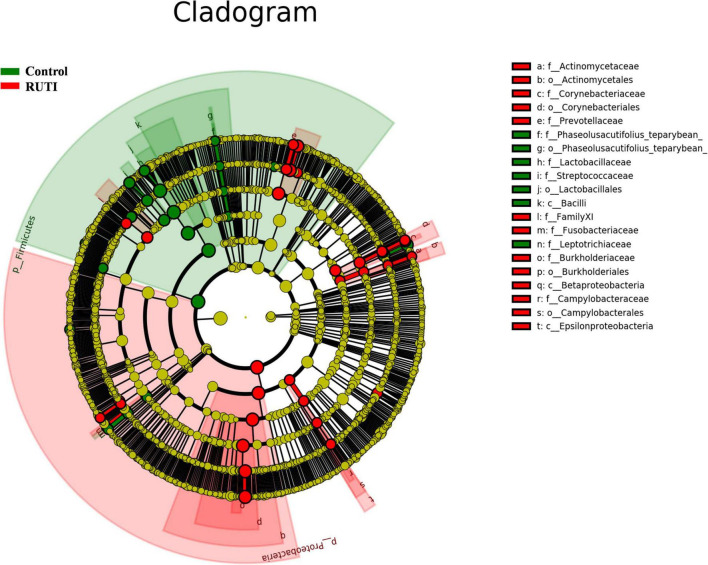
Cladogram representation of differentially abundant taxonomic clades with LDA score > 3.0 in RUTI and controls. In cladogram, the circles radiating from inside out represented taxonomic levels from phylum to genus. Each small circle represented a classification at different levels. The diameter of small circles was proportional to its relative abundance. Those classifications with no significant difference were marked yellow. Red and green represented RUTI and control groups.

In the multivariate statistical analysis, LDA (LEfSe) was used to analyze the differentially abundant taxa as taxonomic biomarkers of RUTI. The relative abundance of 36 bacterial taxa was significantly higher, and another 24 bacterial taxa were significantly lower in RUTI compared with control (LEfSe analysis, *P* < 0.05, LDA score > 3). Specifically, *Proteobacteria*, *Burkholderiales*, *Ralstonia*, *Prevotella*, *Dialister*, and *Corynebacterium* were more abundant in RUTI, while *Lactobacillus*, *Gardnerella*, Viridans *Streptococci*, and *Ezakiella* were significantly reduced in the RUTI group (Figure 5). Cladogram displayed differentially abundant taxonomic clades with an LDA score of >3.0 between RUTI (red) and control (green) groups (Figure 6), which was consistent with the LEfSe result.

## Discussion

The previous study has showed that 94.1% of patients with clinically diagnosed pyelonephritis were treated with antibiotics empirically, but only 55.0% of them had positive urine culture; thus, the diagnostic uncertainty led to inappropriate antibiotic usage (Shallcross et al., 2018). The SUC technique was originally designed in 1956 with the threshold of urinary bacterial count ≥ 10^5^ CFU/ml (Kass, 1956, 1957). However, concerns about the suitability of this threshold for different kinds of UTI (e.g., urethritis, cystitis, and pyelonephritis) were raised (Stamm et al., 1982; Sathiananthamoorthy et al., 2019). Naboka et al. (2020) showed *Enterobacteriaceae* from the urine of women patients with RUTI at 10^2^ CFU/ml had a wide spectrum of virulence factor genes, which may play a role in RUTI. However, the bacteria of 10^2^ CFU/ml could not be detected by SUC. More sensitive methods are required. Current standards for UTI diagnosis had limitations and reduced the opportunity to improve patient care. Thus, new methods and technologies combined with rigorous clinical research could be used to correct the limitations (Price et al., 2018). NGS for bacterial 16S rRNA gene is more sensitive and can detect more atypical bacteria, anaerobes, or mixed pathogens causing UTI compared to urine culture (McDonald et al., 2017). NGS was applied in the diagnosis of chronic or persistent lower urinary tract symptoms (LUTS) and indicated that urinary microbiota and dysbiosis were possibly involved in LUTS. It may affect patients’ clinical outcomes in a variety of clinical scenarios (Gasiorek et al., 2019).

Since there was no standard protocol for the collection and extraction of bacterial DNA from urine, we followed a previous study (Gasiorek et al., 2019) with a larger volume of urine for DNA extraction to get a better detection rate. About 75% of patients with RUTI and 71% of asymptomatic controls were successfully amplified and sequenced the 16S rRNA gene. Other unsuccessfully amplified samples were repeated for a second round. Those still unsuccessful amplified samples after the second round were considered to be non-detected/negative. The previous study has showed that the detection rates of NGS in urgency urinary incontinence (UUI) and non-UUI urine samples were 63.9 and 65.8%, respectively (Pearce et al., 2014), which was lower than our study. Pearce et al. (2014) sequenced 1 ml of catheterized urine. Our study sequenced 30 ml of voided urine. Thus, our study detected bacteria in a greater percentage of samples. Another study found that bacterial DNA was detected in 64.9% of urine samples by 16S rRNA gene sequencing, while about 90% of them were negative by SUC (Hilt et al., 2014; Pearce et al., 2014). Our results confirmed their findings that SUC may miss some low biomass or slow-growing bacteria in patients with RUTI, which could be detected by 16S rRNA gene sequencing.

The alpha diversity index of chao1 and observed species showed that the RUTI group was less diverse than asymptomatic controls, possibly due to previous antibiotic usage, leading to reduced microbiota diversity or dysbiosis. The antibiotic could affect the gut microbiota, leading to loss of taxonomic and functional diversity (Lange et al., 2016). Another possibility could be antibiotics came after the first dysbiosis and symptomatic episode, and then antibiotics exacerbated dysbiosis. Beta diversity of PLS-DA analysis showed that the individuals in the RUTI and control groups were mainly in two different regions, as shown in Figure 4 (ANIMORM, *P* < 0.05). The scattered subjects may be due to individual difference, as hormonal status, body mass index, and certain clinical conditions may to some extent affect women’s urinary microbiota (Mueller et al., 2017).

Yoo et al. (2021) compared the microbiota of women with acute uncomplicated cystitis (AUC) and recurrent cystitis (RC). They found that most AUCs were caused by a specific pathogen. However, various species other than *Enterobacteriaceae* were detected in RC (Yoo et al., 2021). Our study showed similar results, suggesting the role of microbiota changes in RUTI. AUC could be detected by SUC, while the circumstances of RUTI/RC were more complex and should be considered to be dysbiosis. The study of RUTI women’s urogenital microbiota is urgently required (Josephs-Spaulding et al., 2021). Thus, our study was performed to compare the women’s urinary microbiota composition with and without RUTI.

Lower thresholds (10^2^–10^3^ CFU/ml) were set for suprapubic puncture urine or transurethral catheterized urine in the guideline of UTI laboratory diagnosis (China Health Professional Standards, 2016). The indications of these invasive procedures include urinary retention and surgery, instead of RUTI. The benefit-to-risk ratio and clinical feasibility of transurethral catheterization are low for patients with RUTI who are always from outpatient/clinic. Thus, they are not pragmatic in many clinical settings, and clean-catch midstream urine is still commonly used in China with a threshold of 10^4^ or 10^5^ CFU/ml (China Health Professional Standards, 2016). Some clinically diagnosed patients with RUTI often got negative urine culture results, which hampered their antimicrobial treatment. Urine samples collected by invasive methods (e.g., suprapubic puncture, and catheterization) are considered to reflect the real circumstance of the bladder, while voided urine may be contaminated by urethral orifice flora. Therefore, the two kinds of urine sample collection may cause a potential difference in NGS sequencing. In this study, we compared the microbiota between RUTI and their age-matched asymptomatic controls that provided baseline levels of normal urinary microbiota. Our results provided the differentiation between RUTI dysbiosis and normal urinary microbiota, which was a real-world study considering the possible contamination of midstream urine. The results had a wider scope of application and universal significance when suprapubic puncture urine or transurethral catheterized urine was unavailable, which was common circumstance in outpatients/clinics in China.

From our list of highly abundant bacteria in RUTI compared with asymptomatic controls, *Corynebacterium*, anaerobes (e.g., *Prevotella*, *Fusobacterium*, and *Anaerococcus*) have been documented to be involved in UTI (Kline and Lewis, 2016; Boyanova et al., 2022). *Ralstonia* was detected in the RUTI group with high relative abundance (Figure 5). It was undetected in controls. *Ralstonia* has been reported to cause a variety of hospital infections, namely, bloodstream infection, osteomyelitis, and meningitis (Ryan and Adley, 2014), and its pathogenic role in RUTI has not been reported yet. Thus, further studies are still needed to verify the pathogenic role of *Ralstonia* in RUTI. *Lactobacillus* and Viridans *Streptococci* are the main components of vaginal microbiota. They were found to be more abundant in asymptomatic controls. These bacteria are possibly commensal and non-pathogenic in RUTI. The previous study (Stapleton, 2016) has also showed the protective role of *Lactobacillus* against UTI. *Gardnerella* has been detected in urinary microbiota previously (Wolfe et al., 2012; Pearce et al., 2014). Our results showed that *Gardnerella* was highly abundant in the control group compared to RUTI (Figure 5). A recent study showed that *Gardnerella* could often be detected in high abundance from midstream urine of asymptomatic European women (Ksiezarek et al., 2021). *Gardnerella* was more frequently detected in UUI microbiota compared with non-UUI (Wolfe et al., 2012; Pearce et al., 2014). Animal study showed that *Gardnerella* in the mouse bladder could activate host pathways necessary for uropathogenic *Escherichia coli* causing RUTI (O’Brien et al., 2021).

Our study had several limitations. First, although 16S rRNA gene sequencing has been used to reveal urinary microbiota in recent studies, it could only identify bacteria well at the genus level and could not detect viruses or fungi. Thus, our results need to be verified by mNGS in future studies. Second, sexual behavior and sexually transmitted infections/diseases could affect urinary microbiota, and we did not have enough information of included patients. Thus, potential confounders could have affected our findings. Third, this is a single-center study located in Beijing, China, and geographical location may affect microbiota to some extent. Thus, future studies including multiple geographic regions are necessary.

## Conclusion

In conclusion, our study reported the pathogen list of RUTI compared with asymptomatic controls, which could guide the hard-to-treat patients with RUTI with negative cultures. The urinary microbiota of women with RUTI with negative SUC was different from age-matched asymptomatic controls, suggesting that the differential urinary microbiota may play an important role in RUTI.

## Data Availability Statement

The data presented in the study are deposited in the repository of National Genomics Data Center of China (http://ngdc.cncb.ac.cn), with the accession number of PRJCA008664.

## Ethics Statement

The studies involving human participants were reviewed and approved by the Ethics Committee of Peking University First Hospital, approval number: 2021-191. The patients/participants provided their written informed consent to participate in this study.

## Author Contributions

LH and XL designed the study and wrote the manuscript. XL and BZ enrolled the participant. LH, DW, and CH collected the data and performed the experiments. PL, LS, and HL analyzed the data. All authors read and approved the final manuscript.

## Conflict of Interest

PL was employed by the Beijing Yitong Qijun Technology Co., Ltd. The remaining authors declare that the research was conducted in the absence of any commercial or financial relationships that could be construed as a potential conflict of interest.

## Publisher’s Note

All claims expressed in this article are solely those of the authors and do not necessarily represent those of their affiliated organizations, or those of the publisher, the editors and the reviewers. Any product that may be evaluated in this article, or claim that may be made by its manufacturer, is not guaranteed or endorsed by the publisher.
